# Editorial: Plasticity of inhibitory cells in health and disease

**DOI:** 10.3389/fnsyn.2022.1127609

**Published:** 2023-01-10

**Authors:** María Clara Gravielle, Leonardo Pignataro, Florence P. Varodayan

**Affiliations:** ^1^Instituto de Investigaciones Farmacológicas (ININFA), Facultad de Farmacia y Bioquímica, Universidad de Buenos Aires, Consejo Nacional de Investigaciones Científicas y Técnicas, Buenos Aires, Argentina; ^2^Office of Academic Affairs - College of Staten Island - City University of New York, Staten Island, NY, United States; ^3^Developmental Exposure Alcohol Research Center and Behavioral Neuroscience Program, Department of Psychology, Binghamton University-SUNY, Binghamton, NY, United States

**Keywords:** GABA_A_ receptor, gamma aminobutyric acid, plasticity, synapse, inhibitory transmission, interneuron

## Introduction

Brain function is dependent on the ability of neural circuits to remodel synaptic connections in response to internal and external stimuli. Historically, brain plasticity studies focused on excitatory synapses, with inhibitory connections considered largely stable. However, there is growing evidence that inhibitory synapses undergo short- and long-term forms of plasticity through a variety of pre- and postsynaptic mechanisms. Activity-dependent regulation of inhibitory transmission by Hebbian mechanisms has been well documented. On the other hand, homeostatic plasticity of inhibitory synapses has been demonstrated under physiological, pathological, pharmacological, and drug abuse conditions. These diverse forms of inhibitory synaptic plasticity represent an important source of neuronal network reorganization, and their crosstalk with excitatory synaptic plasticity maintains the brain's overall excitatory/inhibitory balance. Despite this growing experimental evidence of inhibitory synapse plasticity, several key issues remain.

## Heterogeneity of interneuron and GABA_A_ receptor subtypes

In this collection, two papers by Fish and Joffe and Speigel and Hemmings review the functional diversity of the main classes of cortical and hippocampal interneurons: parvalbumin INs, somatostatin INs, 5HT3a serotonin receptor INs, calretinin/vasoactive intestinal peptide INs and neurogliaform/ivy cells. These articles describe neurophysiological properties, sites of synaptic connections, and expression of molecular targets in these GABAergic cells. They also discuss the different IN plasticity mechanisms induced by anesthetics and alcohol. Garcia DuBar et al. examined the synaptic connectivity of INs within the pontine circuit that coordinates arousal and voiding behaviors associated with micturition. The authors demonstrate that only somatostatin INs contribute to behavioral modulation, supporting the heterogeneity of GABAergic cell populations and indicating that they participate in specific brain circuitry. Finally, by investigating the biophysical and pharmacological properties of the δ-containing subtype of the GABA_A_ receptor, Shu et al. highlight the importance of different GABA_A_ receptor subtypes in inhibitory plasticity. Given the wide variety of INs, the intricacy of their microcircuits, and the importance of GABA_A_ receptor subtypes, more studies are needed to fully elucidate the mechanisms of inhibitory plasticity during development and in adulthood.

## Coordinated plasticity of excitatory and inhibitory synapses

The importance of this topic is highlighted in the review by Melkumyan and Silberman which examines the opposing effects of chronic alcohol on glutamatergic and GABAergic neurotransmission in the central amygdala. These authors introduce new players (astrocytes and microglia) and molecules (TNFα and IL-1β) and conclude that further studies are needed to determine the contribution of all these cell types, receptors and neuromodulators to the balance of excitation/inhibition neurotransmission. Similarly, the articles by Chapman et al. and Tipton and Russek review the interplay of plasticity between excitatory and inhibitory synapses. They describe how the induction of inhibitory LTP causes excitatory LTD and reduces excitatory neurotransmission. Conversely, they explain that stimulation-induced excitatory LTP dampens proximal inhibitory synapses (LTD). Thus, convergence of glutamatergic and GABAergic signaling mechanisms may allow for coordinated receptor plasticity and balancing of excitation/inhibition. They also identify calcium as a master regulator of synaptic crosstalk, and describe the importance of posttranslational modifications of the receptors and scaffolding proteins to coordinate the interplay of glutamatergic and GABAergic synapses. Since the homeostatic balance between excitation and inhibition is important for brain functioning, future studies should explore the imbalance of inhibitory and excitatory synaptic plasticity during disease.

## Pathology of inhibitory synaptic plasticity in the search for novel therapeutics

Homeostatic disruption due to pharmacologically active compounds or drugs of abuse can induce plastic changes in inhibitory neurotransmission, as can long-term pathology. These alterations are associated with diverse neuropsychiatric and neurological disorders. Several papers in this collection address these issues in the context of therapeutic drug discovery. Speigel and Hemmings explain that anesthetics cause temporary cognitive dysfunction in adults and long-term effects in neo- and pre-natal babies due to disruptions in IN plasticity and development. Neonatal propofol exposure causes parvalbumin and somatostatin IN hypoactivity and vasoactive intestinal peptide IN hyperactivity, suggesting differential cellular effects. Similar to anesthetics, varied sensitivity to acute and chronic alcohol exposure has also been observed, as discussed by two reviews in the collection. Melkumyan and Silberman focus on alcohol's effects on distinct sub-regions of the central amygdala. The authors analyze the role of neuroinflammatory cells, the endocannabinoid system and different neuropeptides and neuromodulators, in the modulation of GABAergic and glutamatergic transmission by alcohol. The article by Fish and Joffe examines inhibitory microcircuits of the pre-frontal cortex as potential targets for the treatment of alcohol use disorder. Notably, both of these papers highlight the important of sex differences in alcohol's regulation of inhibitory synaptic plasticity and advocate for greater identification of sex-specific mechanisms that can be targeted for therapeutic drug development.

Altered inhibitory synaptic plasticity has been associated with the cognitive decline that accompanies normal aging and in Alzheimer's disease. Mackenzie-Gray Scott et al. studied the correlation between hippocampal parvalbumin IN-generated gamma rhythmicity and amyloid beta plaques in a mouse model of Alzheimer's disease. The authors found no relation between gamma oscillations and plaque formation suggesting that hippocampal network activity is resilient. In contrast to Alzheimer's disease, the onset of epilepsy can occur during childhood or as an adult. Regardless of its genetic or acquired basis, inhibitory plasticity and shifts in the excitation/inhibition balance contribute to its pathophysiology, as reviewed by Tipton and Russek. The authors outline the plasticity mechanisms that control GABA_A_ receptor function, modulation of inhibitory synapse formation and elimination, and the vulnerability of selective IN classes to seizures. Tipton and Russek highlight the importance of using single cell studies to identify selective IN vulnerability and develop more specific therapeutic approaches. They also propose identifying factors that make these cells resilient to the disease in order to promote survival of susceptible IN populations.

## Concluding remarks

This Research Topic outlines recent progress and future directions in the field of inhibitory synaptic plasticity. We hope this collection will encourage further studies to elucidate the mechanisms of inhibitory transmission regulation, thus contributing to the development of new therapeutic interventions for neuropsychiatric and neurological disorders.

## Author contributions

All authors listed have made a substantial, direct, and intellectual contribution to the work and approved it for publication.

